# E40 glutenase detoxification capabilities of residual gluten immunogenic peptides in *in vitro* gastrointestinal digesta of food matrices made of soft and durum wheat

**DOI:** 10.3389/fnut.2022.974771

**Published:** 2022-09-08

**Authors:** Gianfranco Mamone, Maria Cristina Comelli, Serena Vitale, Luigia Di Stasio, Katharina Kessler, Ilaria Mottola, Francesco Siano, Linda Cavaletti, Carmen Gianfrani

**Affiliations:** ^1^Institute of Food Science, National Research Council of Italy, Avellino, Italy; ^2^Nemysis Limited, Dublin, Ireland; ^3^Institute of Biochemistry and Cell Biology, National Research Council of Italy, Naples, Italy; ^4^Fondazione Istituto Insubrico Ricerca per la Vita, Varese, Italy

**Keywords:** coeliac disease (CeD), gluten immunogenic peptides (GIP), gliadins, oral enzymatic therapy (OET), Endoprotease 40 (E40)

## Abstract

Gluten degrading enzymes, which are commonly referred to as “glutenases,” represent attractive candidates for the development of a pharmacological treatment of gluten related disorders, such as coeliac disease (CeD). Endoprotease-40 (E40), a novel glutenase secreted by the actinomycete *Actinoallomurus* A8 and recombinantly produced in *S. lividans* TK24, was shown to be active at pH 3 to 6 (optimum pH 5), resistant to pepsin and trypsin degradation, able to destroy immunotoxicity of both gliadin 33-mer peptide and whole proteins and to strongly reduce the response of specific T cells when added to gliadin in *in vitro* gastrointestinal digestion. This study aims to functionally assess the capabilities of Endoprotease-40 (E40) to detoxify residual gluten immunogenic peptides in gastrointestinal digesta of food matrices made of soft and durum wheat. The INFOGEST harmonized protocols were applied to the multicompartmental model of simulated human gastrointestinal digestion, for the quantitative assessment of residual gluten in liquid (beer) and solid (bread and pasta) foods, made of either soft or durum wheat. Proteomic and immunological techniques, and functional assays on intestinal T cell lines from celiac disease patients were used to identify gluten-derived immunogenic peptide sequences surviving in gastric and gastrointestinal digesta after the addition of E40 at increasing enzyme: wheat proteins ratios. During the gastric phase (2 h incubation time), the addition of E40 demonstrated an extensive (≥ 95%) dose-dependent detoxification of whole gluten in real food matrices. Overall, the residual gluten content was found at, or even below, the 20 ppm gluten-free threshold for soft and durum wheat-based food. Furthermore, unlike in untreated gastrointestinal digesta, none of the immunodominant α-gliadin peptides survived in E40-treated digesta. Traces of ω- and γ-gliadin derived immunogenic peptides were still detected in E40-treated digesta, but unable to stimulate celiac-intestinal T cells. In conclusion, E40 is a promising candidate for the oral enzymatic therapy of CeD, as a stand-alone enzyme being efficient along the complete gastrointestinal digestion of gluten.

## Introduction

The worldwide prevalence of diet-related diseases has been on the increase for the last few decades. Coeliac disease (CeD) is a chronic, gluten-driven autoimmune gastrointestinal illness affecting individuals with a predisposing human leukocyte antigen (HLA)-DQ2 and/or -DQ8 genotype (1). Estimated prevalence of the disease is at least 1% in the general population, although many affected patients remain undiagnosed (2). In humans, gastric, duodenal and brush border membrane proteases are unable to fully cleave the intra-chain bonds of glutamine- and proline- rich motifs present in gluten (3), leading to the release of gluten immunogenic peptides (GIPs). GIPs are known to immunostimulate HLA-DQ2/DQ8-restricted intestinal CD4 + T cells, for which gluten epitopes have been mapped (4). In particular, *in vitro* studies with T cells isolated from jejunal mucosa or peripheral blood of CeD patients demonstrated that following deamidation by tissue transglutaminase (tTG), glutamine and proline enriched GIPs enhance their capability to stimulate adaptive immune response in CeD patients (5–9). Accidental or inadvertent gluten exposure, caused by traces of gluten, can be injurious in approximately half of CeD patients, despite their adherence to a strict life-long gluten-free diet (GFD). There is a demand for effective therapeutic adjuncts, or alternatives to the strict GFD (gluten in food ≤ 20 ppm, corresponding to 20 mg/kg, as per Codex Alimentarius), that to date represents the mainstay of treatment for CeD. Nevertheless, GFD is difficult to maintain and can lead to social isolation because of widespread use of gluten in modern diets (10, 11).

Bacterial- plant- or fungal-derived enzymes that are able to digest the immunogenic peptides of gluten and render them non-toxic are commonly referred to as “glutenase” (12). These gluten-degrading enzymes have been proposed as a viable “Oral Enzymatic Therapy” (OET) to assist the GFD (13). Glutenases used for this purpose, including prolyl endopeptidases, cysteine proteases and subtilisins, originate from environmental species of bacteria, fungi, plants, and a few of them have been *in vitro* engineered to increase their glutenase activity, particularly in the gastric environment (13). Glutenases have to be resistant to gastric pH and to human digestive enzymes, thereby remaining active in gastric digestive conditions, with the aim of preventing CeD-specific immune reactions by residual gluten peptides reaching the small intestine. The development of glutenases is based on the clear understanding of GIPs generated during gluten-containing food digestion, with particular reference to the immunodominant α-gliadins peptides indeed. α-gliadins derived GIPs are immunostimulatory in almost all CeD patients, whereas ω- and γ-gliadins and glutenins are less frequently toxic (4, 6, 9). Moreover, GIPs detection in stool and urine are clinically relevant biomarkers of GFD adherence or gluten exposure, and it’s increasingly in practice to confirm the mechanism of action of an OET (14).

The novel microbial Endoprotease 40 (E40, Nemysis Ltd.) was recently discovered as a secreted protein from the soil actinomycete *Actinoallomurus* A8, and its recombinant active form is produced by *Streptomyces lividans* TK24 (WO2013083338; WO2021013553) (15). It has been previously shown to break down the most immunogenic 33-mer, as well as the whole gliadin proteins, strongly reducing the release of interferon (IFN)-γ when exposed to T cells isolated from CeD patients. E40 was previously reported to be maximally active at pH between 4 and 6 and is completely resistant to digestion by gastric and duodenal proteases (15).

Here, we investigated E40’s capability to degrade gluten in real solid and liquid food matrices, such as pasta from *Triticum durum* wheat, bread from *Triticum aestivum* soft wheat, and beer from barley and wheat malt. The *in vitro* static multicompartmental INFOGEST model (16, 17) was applied to digest gluten, because of the recently reported, excellent correlation in protein degradation, and mostly in peptide patterns, between experimental digesta and human jejunum digesta (18). Following its addition to the gastric phase, E40’s dose-dependent glutenase properties were assessed. Assessment was done on two gluten fractions: the fraction of gluten that was soluble (i.e., extracted) in the simulated gastric and intestinal fluids used in the INFOGEST model, and the fraction that remained insoluble (i.e., entrapped in solid remnant food particles). A multi-omics approach based on proteomics, ELISA, and functional assays in human CeD DQ2-restricted T-cell lines (iTCLs), was employed to assess the pattern of residual GIPs in control and E40 gastric (G) and gastrointestinal (GI) digesta. The results provide a promising basis to establish whether the observed conditions are achievable end-points in the clinical setting, thus confirming E40 as a leading candidate for the OET of CeD.

## Materials and methods

### Food matrices and measurement of total protein and gluten content

Bread was prepared at Institute of Food Science (ISA) laboratory, using Italian soft wheat flour (*Triticum aestivum* cv. *Mieti*) and baker’s yeast as leavening agent (19). Dough was kneaded and cooked according to canonical recipe. Pasta spaghetti (durum wheat; Barilla brand), and wheat-beer (*Paulaner Weissbier* brand) were purchased at a local market. Gluten protein concentration in food samples was extrapolated from total protein content (nitrogen × 6.25) measured with the Kjeldahl’s method, the regulatory European and United States accredited standard method for protein quantification in foodstuffs (20), accounting for 80% of the total protein content (21). Experiments were performed in triplicates.

### E40 enzymatic units (EU) calculation

Lyophilized formulated and powdered E40 (F59/60 batch; Maltodextrin 16.5–19.5 DE as excipient) was provided by Nemysis Ltd. Enzymatic Units added to digesta were calculated by the Standard Activity Assay on the substrate N-succinyl-Ala-Ala-Pro-Phe-p-Nitroanilide at 37°C in citrate/disodium phosphate buffer pH 5.0, as described in (15), except that enzyme samples were solubilized in dd-water (specifically, E40 powder batch at 10 mg/ml), then diluted with 20% ethanol to reach the 1–5 nM final E40 concentration in the reaction well (2% final EtOH). Reaction products were monitored spectrophotometrically at 410 nm; standard product curve was determined in the same experimental conditions. A unit of E40 is defined as the quantity of enzyme that releases 1 μmol of *p*-nitroaniline per min, under the specified conditions (Supplementary Figure 1).

### *In vitro* static multicompartmental model of digestion and INFOGEST protocols

*In vitro* digestion included sequential oral, gastric and duodenal phases, using simulated salivary (SSF), gastric (SGF) and intestinal fluid (SIF) respectively, in agreement with the validated INFOGEST methods (16, 17). All digestion steps were carried out in shaking incubator at 170 rpm, at 37°C. Crumb bread samples (5 g) underwent *in vitro* digestion. Pasta samples (5 g) were cooked following the vendor’s package instructions, in boiling salted water. To mimic human oral chewing at meal, bread and pasta samples were grossly minced using a common manual mincer, and not homogenized. Samples were then suspended in 10 ml of SSF (including human salivary amylase at 97.8 U/mg solid minced food) and incubated for 2 min. A beer sample (5 ml) was directly diluted into SGF solution, omitting the oral phase. In the gastric phase, the bolus was diluted with 10 ml SGF including 0.5 ml of phospholipids (10 mg/ml). The gastric pH was adjusted to 4.5 to mimic the physiologic fed condition (22–24) with HCl, and 0.5 ml porcine pepsin (3,839 U/mg solid bolus) was added at a concentration of 12 mg/ml. For the E40-assisted digestion, but not for control samples, E40 was added to the gastric phase at a E40: substrate (w:w) ratio of 1:20, 1:50, and 1:100, with substrate meaning the whole protein food content, also including gluten (Supplementary Table 1). The gastric phase lasted 2 h at 37°C.

For the duodenal phase, the chyme was diluted with 20 ml of SIF and the pH raised to 7.0 with 1 mol/L NaOH. Duodenal digestion was carried out with two different protocols, in agreement with INFOGEST methods. SIF including bile salts (10 mmol/L in the final mixture, measured as cholic acid) was added to pancreatin, the amount of which was calculated on the basis of trypsin activity (7.22 TAME, i.e., trypsin units/mg pancreatin), giving 100 U trypsin/ml final volume. Alternatively, individual enzymes were added to the chyme: bovine α-chymotrypsin (25 U/ml), porcine trypsin (100 U/ml TAME), pancreatic α-amylase (200 U/ml) and pancreatic lipase (2000 U/ml). For both duodenal protocols, the samples were incubated for 2 h at 37°C, and the reaction stopped by boiling in a water bath at 100°C for 5 min (16, 17).

All samples (E40-assisted digestion samples and control samples) were assessed at two time points: (i) after the gastric phase (2 h; referred to as gastric (G) digesta) and, (ii) at the end of the duodenal phase [referred to as gastrointestinal (GI) digesta, i.e., an additional 2 h at neutral pH, for a total of 4 h gluten digestion]. In order to inactivate all the enzymes, the digested samples were boiled soon after the 2 h of digestion to assess the gastric phase, whilst for the experiments to assess the intestinal digestion, the samples were boiled after the additional 2 h of intestinal phase.

Gastric and GI digesta were centrifuged at 10,000 rpm for 30 min, and digested solubilized gluten (supernatants) was separated from the pellet (insolubilized gluten). Digesta were aliquoted at 1 ml, and made salt free by passing through by C18 cartridge Sep-Pak (Waters). Desalted soluble fractions and insoluble fractions were stored at −80°C until use.

### Immunological competitive- and sandwich-R5 ELISA for the quantitation of residual gluten immunogenic peptides in digesta

Residual gluten content in gastric (G) and gastrointestinal (GI) digesta was assessed by R5 ELISA (monoclonal antibody assay, RIDASCREEN^®^ Gliadin Assay, R-Biopharm, Germany), specifically developed for the quantitative analysis of residual gluten in food (25, 26). According to manufacturer’s instruction, the quantification ranges were 5–80 ppm and 5–270 ppm for R5 sandwich and R5 competitive, respectively. Residual GIPs immunogenicity was detected in soluble (R5 competitive) and insoluble (R5 sandwich) G and GI digesta samples. For R5 competitive assay, 1 ml of digesta (without desalting phase) was analyzed. For R5 sandwich, the gluten fraction remaining entrapped in the insoluble fraction was first extracted with cocktail buffer (R-Biopharm). Each sample was tested in triplicate. Assays were carried out according to the provider’s instruction. In our experimental conditions, the limit of detection (LOD) was 5 ppm for both R5 ELISA methods.

### LC-MS/MS analysis of immunogenic gluten-derived peptide sequences

Gastrointestinal digesta (GI, i.e., gastric + duodenal digesta for a total of 4 h digestion time) were assessed by Q Exactive Orbitrap mass spectrometer (Thermo Scientific), coupled online with an Ultimate 3,000 ultra-high performance liquid chromatography instrument (Thermo Scientific, MA, United States). Desalted soluble -gastrointestinal digesta were suspended in 0.1% (v/v) formic acid solution, loaded through a 5 mm long, 300 mm i.d. pre-column (LC Packings, United States) and separated by an EASY-Spray™ PepMap C18 column (25 cm × 75 μm) with 2 μm particles and 100-Å pore size (Thermo Scientific, MA, United States). Eluent A was 0.1% formic acid (v/v) in Milli-Q water; eluent B was 0.1% formic acid (v/v) in acetonitrile. The column was equilibrated at 4% B. Peptides were separated applying a 4–40% gradient of B over 60 min. The flow rate was 300 nl/min. The mass spectrometer was operated in data-dependent mode and all MS1 spectra were acquired in positive ionization mode with an m/z scan range of 300–1,600. Up to 10 most intense ions in MS1 were selected for fragmentation in MS/MS mode. A resolving power of 70,000.00 full width at half maximum (FWHM), an automatic gain control (AGC) target of 1/106 ions and a maximum ion injection time (IT) of 256 ms were set to generate precursor spectra. MS/MS fragmentation spectra were obtained at a resolving power of 17,500.00 FWHM. In order to prevent repeated fragmentation of the most abundant ions, a dynamic exclusion of 10 s was applied. Ions with one or more than six charges were excluded.

LC-MS/MS data were analyzed by Max-Quant software (version 2.0.3.0). The searches were taxonomically restricted to *Triticum* gliadin (Taxonomy 4564) downloaded from UniprotKB database in September 2021. Searching parameters were the following: mass tolerance value 20 ppm for the precursor and 0.05 for the fragment ions. Peptide Spectrum Matches (PSMs) were filtered using the target decoy database approach at 0.01 peptide-level false discovery rate (FDR), corresponding to a 99% confidence score, and validation based on the *q*-value. Statistical analysis was then performed in the Perseus software (version 1.6.15.0). To compare peptide profiles of different digested food sample, MS/MS intensity values were log2-transformed and the three technical replicates grouped. For further analysis, only peptides with two valid values in the groups were included. Missing values between replicates were imputed to normal distribution (width = 0.3, shift = 1.8) (< 1 × 10^–5^).

### Generation of gliadin-specific intestinal T cell lines and interferon-γ release upon digesta challenge

Jejunal biopsies were obtained from *N* = 8 HLA DQ2.5/2.2 CeD patients (mean age 18, range 2 – 40 years). Four pediatric patients (three with overt-CeD, one with potential-CeD) were enrolled at the Department of Translational Medical Science, Section of Pediatrics, University of Naples Federico II (Ethical Committee Prot No 113/2017 and Prot No 347/2017). Four adult patients (two with overt-CeD and two with treated-CeD) were enrolled at the Unit of Gastroenterology, Moscati Hospital, Avellino, Italy (Ethical committee Registry Nos. 16882 and CECN/819). All patients gave their full informed consent to the study. Primary gliadin-reactive iTCLs were generated from jejunal biopsies, as previously described (27–29). Briefly, mucosal explants were digested with collagenase-A for 1 h under a gentle stirring. Detached cells were suspended at 3–5 × 10^5^/ml in complete medium (X-Vivo15 supplemented with 5% AB + human serum and antibiotics, all provided by Lonza, Belgium). Intestinal cells were stimulated with 1.5 × 10^6^ irradiated peripheral blood mononuclear cells (PBMCs), as antigen presenting cells and deamidated enzymatic digest of gliadin (dPT-gliadin, 50 μg/ml). IL-15 and IL-2 (R&D System, Minneapolis, MN, United States) was added after 48 h at 10 ng/ml and 10 U/ml, respectively. On day 7 and 21, iTCLs were re-stimulated with irradiated PBMCs and dPT-gliadin. The immunostimulatory activity of GIPs in gastric and gastrointestinal digesta was performed by co-incubating CeD iTCLs (3 × 10^4^) with allogenic HLA-DQ2.5-positive immortalized B-cells (B-LCLs), as antigen presenting cells (1 × 10^5^), in complete medium (200 μl/well). All untreated and E40-treated digesta were deamidated with tTG and assayed at 50–100 μg/ml concentration. After 48 h, IFN-γ production was measured on cell supernatants by sandwich ELISA (20–22), in duplicates. Purified and biotin-conjugated anti-IFN-γ MoAbs were purchased from Mabtech (Nacka Strand, Sweden). iTCLs from each CeD patient was assayed against each digesta in at least three separate experiments.

### Statistical analysis

GraphPad Prims 6 was used for statistical analysis. One-way ANOVA with Dunnett’s test was used to compare E40-treated groups to the shared control group. One-way ANOVA followed by *post hoc* Bonferroni tests was applied for the intra-group comparison (e.g., control gastric vs. control gastrointestinal digesta, E40 1:20 gastric vs. E40 1:20 gastrointestinal digesta, etc.). A *p*-value of < 0.05 was considered statistically significant. Data are shown as mean ± SD, unless stated otherwise.

## Results

### *In vitro* food digestion

Foods were digested according to the INFOGEST protocol (16, 17), with E40 added to the gastric phase. In order to establish the enzyme (E40): substrate (proteins) ratio, the protein concentration of the foods was first evaluated. Bread, pasta and beer showed a total protein content (g protein/100 g food; mean ± SD) of 10.8 ± 0.6, 12.2 ± 0.5, 1.13 ± 0.15 of dry samples, respectively. Gluten content corresponded to 80% of total protein content (21), leading to 432, 488, and 45.2 mg gluten in the 5 g of bread, pasta, and beer to be digested. The gluten content was measured on the uncooked pasta sample as, unlike bread, the cooking process of pasta may induce a leak of some proteins in the cooking water which are mainly albumin and globulin fractions, while gluten proteins are not affected (30).

**FIGURE 1:**
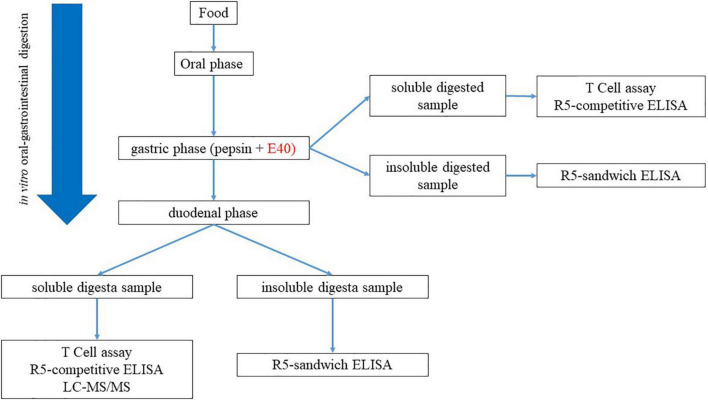
Experimental workflow for the assessment of GIPs in gastric and gastrointestinal; digesta.

Residual GIPs in gastric (G), and gastrointestinal (GI) digesta were assessed by proteomics and immunoassay. Figure 1 exhibits the schematic diagram of the experimental design.

### Quantification of residual gluten in digesta

**FIGURE 2:**
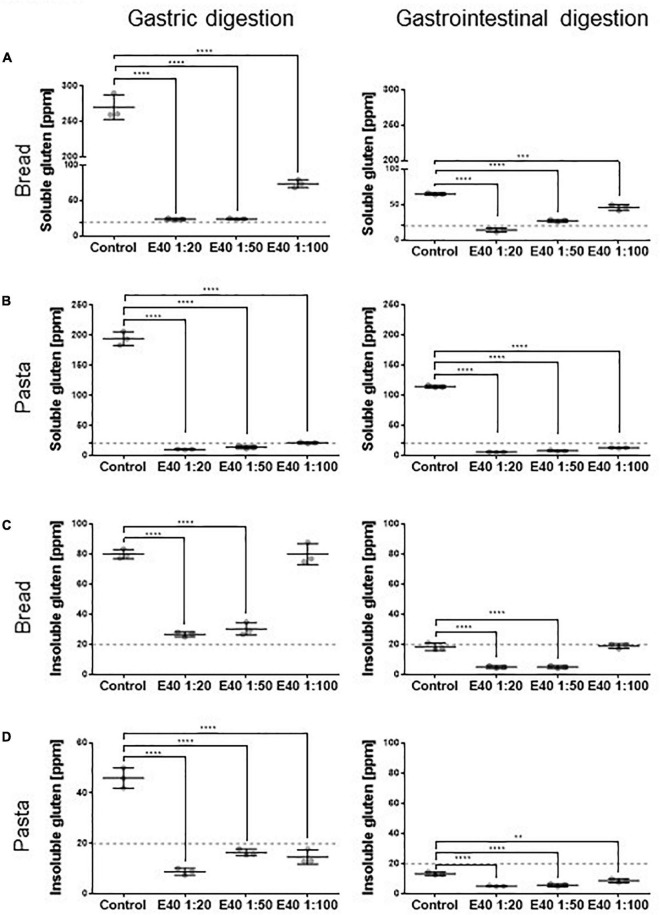
Residual soluble and insoluble gluten content in bread **(A,C)** and pasta **(B,D)** samples. **(A,B)** Residual gluten content in its soluble form as assessed by competitive R5 ELISA. **(C,D)** Residual gluten content in its insoluble form as assessed by sandwich R5 ELISA. Dotted line: 20 ppm, the threshold for gluten-free food. Data are shown as mean ± SD of *n* = 3 repetitions. Data were analyzed by one-way ANOVA followed by Dunnett’s multiple comparisons test, *: 0.01 < *p* < 0.05; **: 0.001 < *p* < 0.01; ***: 0.0001 < *p* < 0.001; ****: *p* < 0.0001.

Figure 2 shows the quantitative assessment of residual gluten in G and GI digesta from bread and pasta samples. The soluble sample, extracted form during digestion, was analyzed by R5 competitive ELISA, while the insoluble fraction, entrapped in remnant pelleted food, was analyzed by R5 sandwich ELISA. E40 shows a robust dose-dependent proteolytic degradation of soluble gluten in soft wheat bread and durum wheat pasta samples after both 2 h gastric (Figures 2A,B, left panel) and 4 h gastrointestinal digestion (Figures 2A,B, right panels). E40 added at a 1:20 ratio to bread samples reduced the residual soluble gluten content to 24.3 and 14.0 ppm, the threshold of gluten-free food, after the gastric and gastrointestinal digestion, respectively (Figure 2A). For pasta samples, the residual soluble gluten content after 2 h gastric and 4 h gastrointestinal digestion was below the 20 ppm gluten-free threshold, at all E40 doses tested (Figure 2B).

E40 shows gastric degrading capability also for the insoluble gluten (Figures 2C,D). At ratios 1:20 and 1:50, E40 reduced the insoluble gluten content of bread to close and below the 20 ppm gluten-free threshold after the gastric and gastrointestinal digestion (Figure 2C), respectively. Added to pasta samples, E40 reduced the residual insoluble gluten content after both gastric and gastrointestinal digestion to well below 20 ppm, at all E40 doses tested (Figure 2D).

Beer samples showed a complete gluten degradation by E40 at all concentrations tested (Supplementary Figure 2, gastric digesta only). As the residual gluten content was already below 20 ppm following gastric digestion, samples of the gastrointestinal digestion were not assessed. The high degradation rate likely arose from a lack of matrix effect in beer.

**FIGURE 3:**
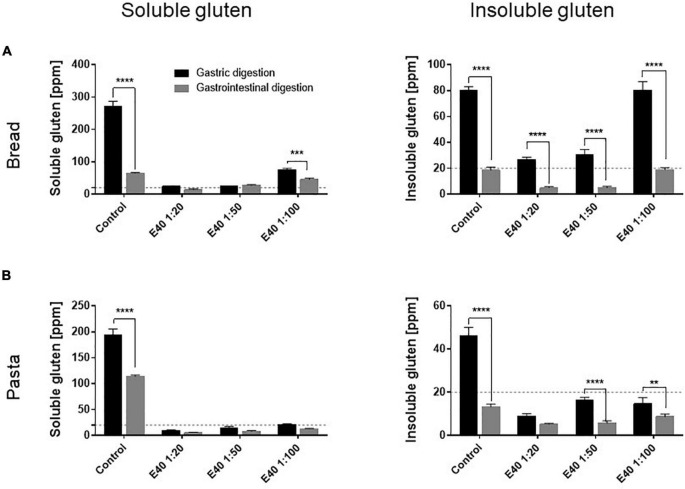
Gluten degrading efficiency in bread **(A)** and pasta **(B)** samples during gastric and gastrointestinal digestion. Left panel: residual gluten content in its soluble form as assessed by competitive R5 ELISA. Right panel: residual gluten content in its insoluble form as assessed by sandwich R5 ELISA. Dotted line: 20 ppm, the threshold for gluten-free food. Data are shown as mean ± SD of *n* = 3 repetitions. Data were analyzed by one-way ANOVA (*p* < 0.0001 for all) followed by *post hoc* Bonferroni tests, *: 0.01 < *p* < 0.05; **: 0.001 < *p* < 0.01; ***: 0.0001 < *p* < 0.001; ****: *p* < 0.0001.

Figure 3 compares the gluten degrading efficacy of the gastric and gastrointestinal digestion, in control and in E40 treated samples. As early as the gastric phase, E40 induced an extensive degradation of soluble gluten at 1:20 and 1:50, down to and even below the 20 ppm threshold of a gluten-free food, for bread (Figure 3A) and pasta (Figure 3B) respectively. Converting enzyme: substrate ratio to E40 mg in assessed digesta, 10.8 mg E40 (as used in the 1:50 ratio) to 27 mg E40 (as used in the 1:20 ratio) proved to extensively degrade the 432 mg (bread) and 488 mg (pasta) gluten contained in the assessed samples, either in its extracted (soluble, Figures 3A,B) as well as in hidden (insoluble, Figures 3A,B) right panel form, showing an overall >90% additional gluten degradation in the extended pH range of 4.5–7.

### Gluten immunostimulatory sequences in digesta

Gluten-derived peptides of bread and pasta were identified by proteomics analysis. Principal component analysis (PCA) and heat maps were plotted to assess differences between GI-control and GI-E40 at all tested concentrations. A total of 310 and 245 peptides from bread and pasta were identified, respectively.

**FIGURE 4:**
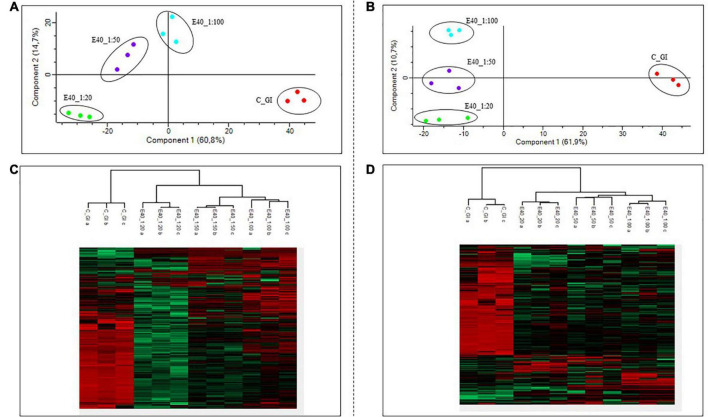
PCA and heat map of all GI peptides identified by LC-MS/MS for bread **(A,C)** and pasta **(B,D)**. **(A,B)** PCA of the technical replicates, reflecting the differences between the resistant peptides of GI control and that of digesta at dose-dependent E40 detoxification (1:20, 1:50, and 1:100). **(C,D)** Heat map of the technical replicates showing the LC-MS/MS intensity of GI-resistant peptides in control samples and digesta with E40 treatment. Red color and green colors represent high and low intensity, respectively; values are scaled across columns, generating column z-scores. Identified sequences are reported in Supplementary Table 2.

The comprehensive list of all residual peptides is provided in Supplementary Table 2. Significant differences in the peptide pattern between control and E40 digesta are visualized using heat maps (Figures 4C,D; bread, pasta), where each row represents one differentially abundant peptide and each column represents one biological replicate. PCA (Figure 4) showed that the three replicates of bread (panel A) and pasta (panel B) were tightly clustered together and food samples digested by E40 were well-separated from control, meaning that digesta are clustered by the effect of E40 dose-dependent detoxification (1:20, 1:50, and 1:100).

**FIGURE 5:**
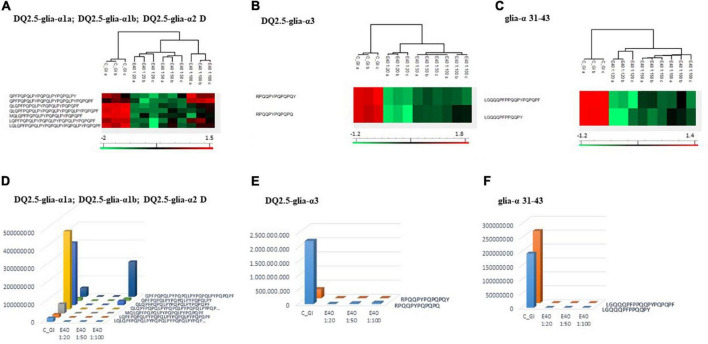
Residual intestinal immunogenic alpha-gliadin CeD sequences in bread control (GI) and digesta at dose-dependent E40 detoxification (1:20, 1:50 and 1:100). **(A–C)** Heatmap of LC-MS/MS analysis of the proteases resistant peptides harboring alpha-gliadin CeD epitopes, of the technical replicates of Control (GI) and E40 glutenase groups; the red color represents high-resistant, and the green color represents low-resistant gluten peptides; values are scaled across columns, generating column z-scores. **(D–F)** Graphical representation of the sum (average value of technical triplicates) of LC-MS/MS intensity of peptides harboring CeD alpha-gliadin epitopes identified in control (GI) and E40 (1:20, 1:50, and 1:100) treated bread sample.

**FIGURE 6:**
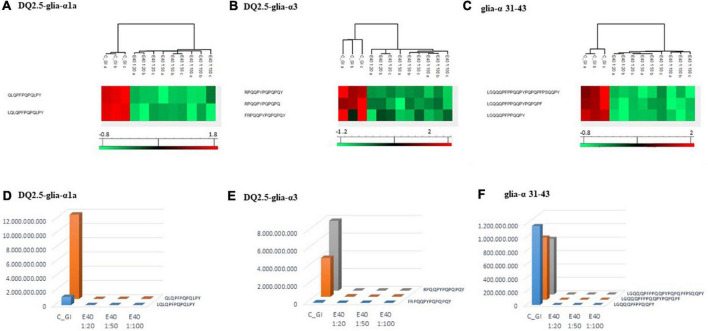
Residual intestinal immunogenic α-gliadin sequences in pasta control (GI) and digesta at dose-dependent E40 detoxification (1:20, 1:50, and 1:100). (**A–C)** Heatmap of LC-MS/MS analysis of the proteases resistant peptides harboring α -gliadin CeD epitopes, of the technical replicates of Control (GI) and E40 glutenase groups; the red color represents high-resistant, and the green color represents low-resistant gluten peptides; values are scaled across columns, generating column z-scores. **(D–F)** Graphical representation of the sum (average value of technical triplicates) of LC-MS/MS intensity of peptides harboring CeD α-gliadin epitopes identified in control (GI) and E40 (1:20, 1:50, and 1:100) treated pasta sample.

Proteomics allowed to identify GIPs harboring immunogenic and/or toxic sequences, and their survival in E40 assisted digestion. Particular attention is given to α-gliadin derived GIPs, because of their high immunotoxicity in almost all CeD patients (9–11). Figures 5, 6 show the heat maps of α-gliadin derived GIPs detected in bread GI digesta (Figures 5A–C) and in pasta GI digesta (Figures 6A–C). Figures 5, 6 also include a graphical representation of E40 induced, complete detoxification of α-gliadin GIPs, at all tested doses (panels D,E). Control GI digesta confirmed the resistance of α-gliadins to gastric and intestinal proteases. Control bread digesta contained the 33-mer (LQPFPQPQLPYPQPQLPYPQPQLPYPQPQPF) peptide, highly resistant to proteases and considered one of the most immunogenic in CeD patients, because it encloses six T-cell overlapping epitopes, consisting of DQ2.5-glia-α1a, DQ2.5-glia-α2 (three copies), and DQ2.5-glia-α1b (two copies). Homologs or shortened forms of 33-mer were also detected (Figures 5A,D). Notably, none of these peptides were detectable in E40 bread digesta at 1:20 and 1:50 dose, whereas fragments harboring these epitopes were identified at 1:100 (Figures 5A,D). Pasta, made of *T. durum* with a tetraploid genome, does not express the 33-mer sequence but a shortened homolog of the 13 N-terminal residues including one copy of the DQ2.5-glia-α1a. In control GI pasta digesta, the 13-mer LQLQPFPQPQLPY sequence was identified together with its counterpart trimmed at N-terminus (12-mer). Notably, none of these peptides were detected in E40 pasta digesta (Figures 6A,D).

Similarly, GIPs harboring the immunogenic epitopes DQ2.5-glia-α3, were identified in control bread and pasta digesta but not in E40 treated samples (Figures 5B–F, 6B–F).

Control bread and pasta digesta also contained the toxic P31–43 peptide (LGQQQFPPQQPY), known to trigger innate immunity in CeD (4), embedded within peptides of different length. Notably, E40 completely prevented the release of these harmful peptides, at all tested concentrations in bread (Figures 5C,F) and pasta (Figures 6C,F), since they were undetectable by LC-MS/MS.

A high number of ω - and γ –gliadin derived peptides, harboring the DQ2.5-glia-ω2, DQ2.5-glia-γ2, and DQ2.5-glia-γ4c epitopes were detected in control bread digesta (Supplementary Figure 3). The latter epitope was also found in control pasta digesta (Supplementary Figure 4). These peptides were mainly released from digestion of γ-gliadin and ω-gliadin, including different fragments of the same protein region, trimmed at N- and C- terminus. E40 was unable to fully prevent the release of these harmful peptides in E40 GI bread and pasta digesta, at all tested concentrations. E40 GI digesta still contained traces of ω- and γ-generated fragments, including intact epitope peptides. However, these epitopes are known for their weaker immunogenicity when compared to parent α-gliadins (26), and, as detailed below, did not induce the release of INF-γ.

### Interferon-γ secretion by coeliac disease T-cell lines after stimulation with gastric and gastrointestinal bread and pasta digesta

**FIGURE 7:**
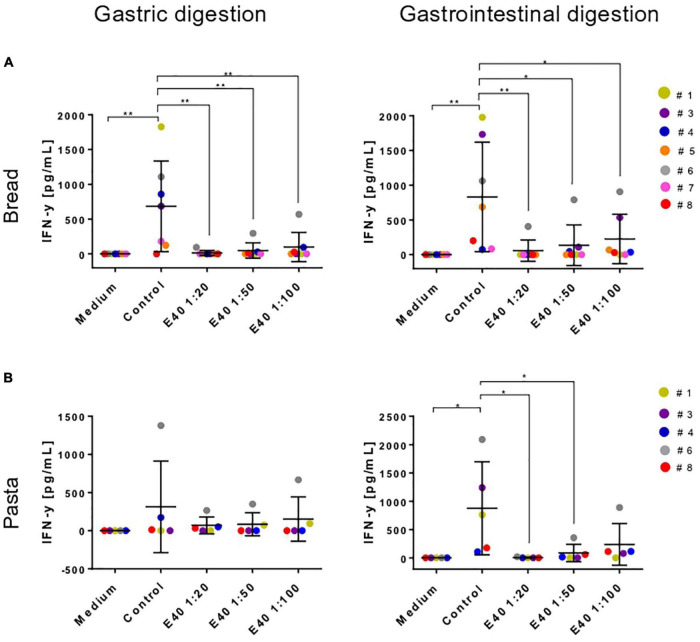
Interferon-gamma secretion by T-cell lines reactive to undigested gluten after stimulation with gastric (left) or gastrointestinal (right) bread **(A)** and pasta **(B)** digesta. IFN-y, interferon-gamma; G, gastric; GI, gastrointestinal. Data are shown as mean ± SD **(A)**: *n* = 7; **(B)**: (*n* = 5 CeD patients). One-way ANOVA followed by Dunnett’s multiple comparisons test was used to analyze the data, *: 0.01 < *p* < 0.05; **: 0.001 < *p* < 0.01; ***: 0.0001 < *p* < 0.001; ****: *p* < 0.0001.

Table 1 details the clinical characteristics of enrolled CeD patients and T epitopes recognition pattern of their derived iTCLs. Figure 7 summarizes the overall IFN-γ release in response to gluten extracts from either control- or E40-treated gastric (G) and gastrointestinal (GI) digesta of bread and pasta samples. A significantly reduced IFN-γ release was observed at all E40 tested doses in E40 gastric (G) bread digesta, with E40 1:20 abating the immunostimulatory response, in that the IFN-γ content was similar to that of the medium of unstimulated cells (Figure 7A, left panel). A further reduction was observed in E40-treated bread GI digesta, even at E40 1:100 (Figure 7A, right panel). Surprisingly, when pasta digesta were assessed for T-cell immunogenicity, a slight although not significant IFN-γ release was observed in response to control G digesta only, when compared to unstimulated cells (medium) (Figure 7B, left panel). Control GI pasta digesta did instead immune-stimulate a significant IFN-γ release. One reasonable explanation for the observed different gastric and gastrointestinal behavior of control pasta digesta is that GIPs are physiologically freed under duodenal digestive conditions, e.g., during the 2 h digestion at neutral pH. A reduced IFN-γ release occurred in all E40-treated pasta samples (both G and GI digesta), and particularly at E40 1:20 and 1:50 in GI pasta digesta (Figure 7B, right panel). Overall, the reduction of immune-stimulated IFN-γ release in CeD iTCLs reactive to immunodominant epitopes of α-gliadin, namely the DQ2.5-glia-α1,2 (Table 1), supports and strengthens the observed extensive α-gliadins detoxification by E40 as seen with proteomics, particularly at 1:20 in bread digesta, and at all tested doses in pasta digesta. A marked ability of E40 to degrade immunogenic sequences in ω- or γ-gliadin was also observed in functional T cell assays. More specifically, although some residual ω- and γ-peptide content are still identified in bread and pasta GI E40-digesta, Supplementary Figure 5 shows a significant reduction of IFN-γ release in CeD iTCLs from patients reacting to ω- or γ- gliadin T epitopes (pt#6 and pt#8, respectively), in response to E40 treated samples vs. untreated. These functional data could suggest that the residual ω- and γ-gliadin peptide content is of no immunogenic concern, in the given experimental conditions.

**TABLE 1:** Enrolled celiac patients for functional T-cell experiments and gluten peptide specificity.

Patient	Age/sex	Diagnosis	Peptide specificity*	Gluten protein	Gluten epitope sequence**
#1	34/F	overt-CeD	DQ2.5-glia-α1,2	α-gliadin	PFPQPQLPY, PQPQLPYPQ
#2	40/F	overt-CeD	Nd	nd	nd
#3	18/M	treated-CeD	DQ2.5-glia-α1,2	α-gliadin	PFPQPQLPY, PQPQLPYPQ
#4	2/F	overt-CeD	DQ2.5-glia-α1,2	α-gliadin	PFPQPQLPY, PQPQLPYPQ
			DQ2.5-glia-ω1,2	ω-gliadin	PFPQPQQPF, PQPQQPFPW
			DQ2.5-glia-γ2	γ-gliadin	IQPQQPAQL
#5	2.5/F	potential-CeD	DQ2.5-glia-α1,2	α-gliadin	PFPQPQLPY, PQPQLPYPQ
			DQ2.5-glia-ω1,2	ω-gliadin	PFPQPQQPF, PQPQQPFPW
#6	5/F	overt-CeD	DQ2.5-glia-ω1,2	ω-gliadin	PFPQPQQPF, PQPQQPFPW
#7	36/F	treated-CeD	DQ2.5-glia-α1,2	α-gliadin	PFPQPQLPY, PQPQLPYPQ
			DQ2.5-glia-ω1,2	ω-gliadin	PFPQPQQPF, PQPQQPFPW
			DQ2.5-glia-γ1	γ-gliadin	PQQSFPQQQ
#8	6.7/M	overt-CeD	26-mer (DQ2.5-glia-γ3,4,5)	γ-gliadin	QQPQQPYPQ, QQPQQPFPQ, PQPFPQQPQ

*Gluten peptide nomenclatures is according Sollid et al. (4). **The glutamine residues target of tTG deamidation are underlined (Q). Nd, not detected.

## Discussion

A deep understanding of the capability of food proteins to stimulate an immune response cannot be separated from the physiological digestive process by proteases of the upper gastrointestinal tract (16, 17, 31).

Gluten proteins have the peculiarity to be highly resistant to hydrolysis by gastrointestinal proteases, due to the high content of glutamine (30–35%) and proline (10–15%) residues (29, 32). This resistance to physiologic digestion ensures the survival in the gut lumen of long peptides with immunogenic potential that reach high concentration levels in the gut epithelium, stimulating either adaptive or innate immune responses in CeD patients. Glutenases are naturally or engineered enzymes highly efficient to degrade gluten proteins (13, 33–35).

In this study, we assessed the capability of E40 to significantly modify the pattern of GIPs generated during the digestion of food matrices by using a validated static *in vitro* digestion model (15, 16). Gluten detection in food is challenging, mainly due to the intercalation of disulfide linked polymeric glutenins with single gliadin molecules in a complex protein network favoring their chemical/physical interactions with other food ingredients, as well as due to technological food processes inducing modifications on gluten proteins (36). Based on that, the standardized INFOGEST protocol was applied to grossly minced gluten-based food matrices, in particular to bread slices and pasta, and to a liquid food like beer. Food digestion was initially carried out according to Minekus et al. (16), based on the use of pancreatin and bile into the duodenal phase (16). However, when coupling digested food with immunochemical assay, the excess of bile salts and pancreatin evoked detrimental effects in T-cells leading to unreadable ELISA results (data not shown). Previously, studies reported that the excess of pancreatin and bile acid may interfere with cell-based experiments (37, 38). To avoid artifacts and unspecific cytotoxicity, we therefore adopted the INFOGEST protocol based on the use of selected proteolytic (trypsin and chymotrypsin), lipase and amylase enzymes (16), and excluding bile salts. This approach allowed to quantify residual gluten in digesta and to verify immunotoxicity by ELISA and T cells assays.

Confirming our previous observations referring to its robust gastric and GI-proteases resistance (15), E40 markedly digested gluten proteins in both liquid and solid food matrices already in the gastric phase, to such an extent that none of the most immunogenic α-gliadins survived in soft and durum gluten-based food after digestion. The absence of bile in the duodenal phase because of interferences with the T cell-functional assay, does not invalidate the glutenase activity of E40. Bile acids are known to promote absorption of dietary lipids by solubilizing them in mixed micelles, whereas they do not facilitate the digestion of other nutrients (39). R5 ELISA, LC-MS/MS and functional celiac T-cell assay outcomes were all consistent in demonstrating that E40 induces an extensive gluten degradation, completely detoxifying the most immunodominant sequences. Residual traces of ω- and γ-gliadin peptides containing immunogenic sequences, although still present in E40 treated digesta, did not elicit a significant IFN-γ production, most likely due to a peptide concentration below the T-cell stimulation threshold, in the given experimental conditions. Marti et al. similarly reported destruction of some γ-gliadin epitopes to be considerably less efficient with the addition of *Flavobacterium meningosepticum* PEP to digestion of 3 g wheat gluten flour (40).

In 2002, Shan et al. reported a prolyl endopeptidase (PEP) from *F. meningosepticum* highly efficient in degrading the α-gliadin 33-mer (41). Since this pioneering study, several other glutenases have been described from a variety of sources including fungi (42–44), bacteria (12) and plants (45, 46), as natural as well as engineered proteins (28, 29, 45–47). Although many of these enzymes have been studied for their ability to degrade gluten either *in vitro* and *in vivo*, including phase I and II clinical trials (33, 47, 48), to date none of them is in clinical practice as an efficient and safe adjunct to GFD for CeD treatment. To be a promising drug, a glutenase should have a high specificity for cleaving proline and glutamine enriched gluten sequences and optimal proteolytic activity in the acidic gastric milieu, and more generally, in the gastrointestinal environment. The use of a wide range of different *in vitro* digestive conditions utilized in published studies makes the meaningful comparison of results among different glutenases difficult, particularly in terms of destruction of T cell epitopes and prevention of GIPs generation (40, 44–48). Many of these GIPs are, so far, regarded as a standard for studying gluten exposure in CeD patients, either to confirm adherence to the GFD, or to detect an inadvertent gluten intake, thus fostering the maintenance of intestinal symptoms, and to some extent signs, in CeD patients (49–51). This study investigated the immune detoxifying capability on gluten peptides recognized by HLA-DQ2.5/2.2, the most represented haplotype in CeD. It would be interesting to extend the study to HLA-DQ8 haplotypes, although much less represented in CeD (1, 2).

E40 demonstrated a robust proteolytic activity of solubilized and hidden gluten in the extended pH range of 4.5–7, successfully detoxifying gluten in the simulated stomach, and maintaining its activity in the proximal intestine. These characteristics identify E40 as a stand-alone enzyme active along the whole gastrointestinal digestive process of gluten.

Considerable inter-individual variability in the interval between gluten consumption and GIP excretion in urine has been reported in the clinical setting, in CeD and also in healthy individuals. Whereas renal dysfunction was generally an exclusion criterion in clinical trials, gastrointestinal motor dysfunction was not considered in those studies (50–53), despite the fact that altered gastric emptying and low intestinal transit have been reported in CeD patients (50–53).

E40 mediated degradation of gluten proteins was previously demonstrated to be efficient within 30 min of gastric incubation (15), and RP-HLPC analysis of E40-treated gastric digesta in this study showed gluten disruption in food matrices within 40–50 min (data not shown). In addition, our results show that E40 is active at both the gastric and small intestinal pH range. Such enzymatic properties offer an extension of gluten degradation in the duodenal tract, were pH value is about 7. These characteristics suggest that E40 could be of particular relevance for people with an altered gastric emptying or a slow intestinal transit.

While E40 is not intended to replace the GFD as a primary and sole management for CeD (54), it is envisaged to adjunct the GFD in order to protect against the detrimental effect of a few hundred milligrams to a few grams of gluten in patients with high gluten sensitivity. The reported fact that 40% of CeD patients with persistent villus atrophy have positive CeD serologies despite adhering to a GFD, is suggestive of commonly ongoing inadvertent gluten exposure or high sensitivity to the gluten content of a GFD, and the need to better control it (11, 50). In this complex scenario, our results foster E40 to be shortly clinically tested in CeD patients, as a candidate for an OET and adjunct to a GFD aimed to manage gluten related disorders.

## Data availability statement

The original contributions presented in this study are included in the article/Supplementary material, further inquiries can be directed to the corresponding author.

## Ethics statement

The studies involving human participants, including biopsies and extraction of CeD derived TLCs, were reviewed and approved by the Ethical Committee of SG. Moscati Hospital, Avellino, Italy (Ethical Committee Registry Nos. 16882 and CECN/819), and Department of Pediatrics, University of Naples Federico II of Naples (Ethical Committee Prot Nos. 113/2017 and 347/2017). Written informed consent to participate in this study was provided by the participants’ legal guardian/next of kin.

## Author contributions

GM and CG contributed to the study conception and design. LC was responsible for E40 batch preparation and E40 activity determination in Standard Activity Assay. SV, IM, LD, and FS carried out the acquisition and analysis of data. GM, MCC, and CG were involved in the interpretation of data and drafting of the manuscript. KK performed the statistical analysis and figure drawing. All authors contributed to the critical revision of the manuscript for important intellectual content and approved the final version of the manuscript.
