# Supplementary figures and images for: E40 glutenase detoxification capabilities of residual gluten immunogenic peptides in in vitro gastrointestinal digesta of food matrices made of soft and durum wheat
Source: Front Nutr. 2022 Sep 8;9:974771. doi: 10.3389/fnut.2022.974771 (PMC9493446; doi:10.3389/fnut.2022.974771)

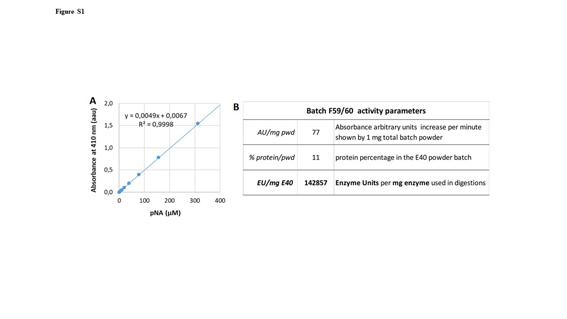

Supplement: Supplementary file 1 [file Image_1.JPEG]

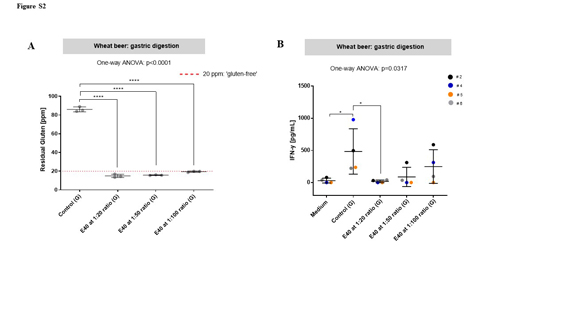

Supplement: Supplementary file 2 [file Image_2.JPEG]

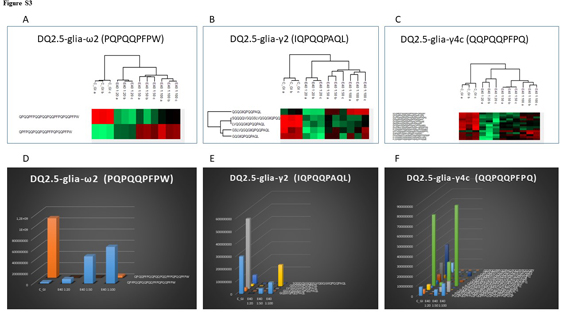

Supplement: Supplementary file 3 [file Image_3.JPEG]

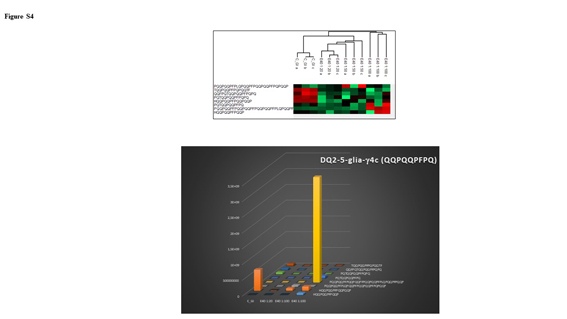

Supplement: Supplementary file 4 [file Image_4.JPEG]

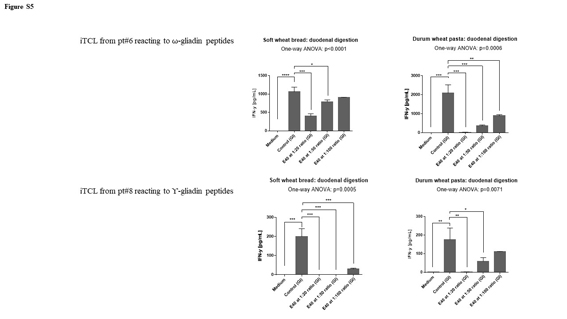

Supplement: Supplementary file 5 [file Image_5.JPEG]
