# Supplementary material for: E40 glutenase detoxification capabilities of residual gluten immunogenic peptides in in vitro gastrointestinal digesta of food matrices made of soft and durum wheat
Source: Front Nutr. 2022 Sep 8;9:974771. doi: 10.3389/fnut.2022.974771 (PMC9493446; doi:10.3389/fnut.2022.974771)
Supplement: Supplementary file 8 [file Data_Sheet_1.DOCX]

**Figure S1**

E40 Enzyme Units determination. (A) Standard para-Nitroaniline curve in the specified assay conditions at pH 5. Absorbance of 1 µmole p-Nitroaniline was calculated according to the extrapolated linear equation. (B) Parameters used to determine Enzyme units per mg of E40. Absorbance arbitrary units produced in 1 min by 1 mg of E40 batch powder in the specified assay conditions (AU/mg pwd) normalized to 1 mg protein (based on BCA method) divided by the absorbance value of 1 µmole p-Nitroaniline measured by A.

**Figure S2.** Residual gluten content (A) and interferon-gamma secretion by T-cell lines (B) following gastric digestion of wheat beer in the presence and absence of E40.

Dotted line: 20 mg/kg, the threshold for gluten-free food. Data are shown as mean ± SD ((A): n=3 repetitions; (B): n=4 CeD patients). Data were analysed by one-way ANOVA followed by Dunnett’s multiple comparisons test. *: 0.01>p>0.05; **: 0.001>p>0.01; ***: 0.0001>p>0.001; ****: p<0.0001.

**Figure S3** Differentially intestinal immunogenic omega- and gamma-gliadin CeD epitopes in bread control (GI) and digesta at dose-dependent E40 detoxification (1:20, 1:50 and 1:100). Panel A, B, C: heatmap of LC-MS/MS analysis of the differentially resistant peptides harbouring omega-gliadin (panel A) and gamma-gliadin (panel B and C) CeD epitopes, of the technical replicates (a, b, and c) of Control (GI ) and E40 glutenase groups; the red colour represents up-resistance , and the green colour represents down-resistance; values are scaled across columns, generating column z-scores. Panel D, E, F: graphical representation of the sum (average value of technical triplicates) of LC-MS/MS intensity of peptides harbouring omega gliadin (panel D) and gamma-gliadin (panel E and F) CD alpha-gliadin epitopes identified in control (GI) and E40 (1:20, 1:50 and 1:100) treated bread sample.

**Figure S4:** Differentially intestinal immunogenic gamma-gliadin CeD epitope in pasta control (GI) and digesta at dose-dependent E40 detoxification (1:20, 1;50 and 1:100). Panel A: heatmap of LC-MS/MS analysis of the differentially resistant peptides harbouring gamma-gliadin CD epitope, of the technical replicates (a, b, and c) of Control (GI ) and E40 glutenase groups; the red colour represents up-resistance, and the green colour represents down-resistance; values are scaled across columns, generating column z-scores. Panel B, graphical representation of the sum (average value of technical triplicates) of LC-MS/MS intensity of peptides harbouring CD gamma-gliadin epitope identified in control (GI) and E40 (1:20, 1:50 and 1:100) treated pasta sample.

**Figure S5**

Interferon-γ production profile in response to GI bread and pasta digesta by intestinal T-cell lines from patients pt#6 and pt#8, reacting to ω-gliadin (PFPQPQQPF, PQPQQPFPW) or γ-gliadin epitopes (QQPQQPYPQ, QQPQQPFPQ, PQPFPQQPQ), respectively (see Table 1). Data are shown as mean ± SD of duplicate INF-γ assessments for each iTCLs. One representative experiment out of three performed for each iTCLs. One-way ANOVA test was used to analyse the data. *: 0.01<p<0.05; **: 0.001<p<0.01; ***: 0.0001<p<0.001.
